# Oriented zinc oxide nanorods: A novel saturable absorber for lasers in the near-infrared

**DOI:** 10.3762/bjnano.9.255

**Published:** 2018-10-23

**Authors:** Pavel Loiko, Tanujjal Bora, Josep Maria Serres, Haohai Yu, Magdalena Aguiló, Francesc Díaz, Uwe Griebner, Valentin Petrov, Xavier Mateos, Joydeep Dutta

**Affiliations:** 1 ITMO University, Kronverkskiy pr., 49, 197101 Saint-Petersburg, Russia; 2 Chair in Nanotechnology, Water Research Center, Sultan Qaboos University, P.O. Box 17, Al-Khoudh, 123 Muscat, Oman; 3 Nanotechnology, School of Engineering and Technology, Asian Institute of Technology, P.O. Box 4, Klong Luang, Pathumthani – 12120, Thailand; 4 Física i Cristallografia de Materials i Nanomaterials (FiCMA-FiCNA)-EMaS, Dept. Química Física i Inòrganica, Universitat Rovira i Virgili (URV), Campus Sescelades, 43007 Tarragona, Spain; 5 State Key Laboratory of Crystal Materials and Institute of Crystal Materials, Shandong University, Jinan 250100, China; 6 Max Born Institute for Nonlinear Optics and Short Pulse Spectroscopy, Max-Born-Str. 2a, 12489 Berlin, Germany; 7 Functional Materials, Applied Physics Department, School of Engineering Sciences, KTH Royal Institute of Technology, Isafjordsgatan 22, SE-164 40 Kista Stockholm, Sweden

**Keywords:** oriented nanorods, Q-switching, saturable absorption, solid-state lasers, zinc oxide

## Abstract

Zinc oxide (ZnO) nanorods (NRs) oriented along the crystallographic [001] axis are grown by the hydrothermal method on glass substrates. The ZnO NRs exhibit a broadband (1–2 µm) near-IR absorption ascribed to the singly charged zinc vacancy V_Zn_^−1^. The saturable absorption of the ZnO NRs is studied at ≈1 µm under picosecond excitation, revealing a low saturation intensity, ≈10 kW/cm^2^, and high fraction of the saturable losses. The ZnO NRs are applied as saturable absorbers in diode-pumped Yb (≈1.03 µm) and Tm (≈1.94 µm) lasers generating nanosecond pulses. The ZnO NRs grown on various optical surfaces are promising broadband saturable absorbers for nanosecond near-IR lasers in bulk and waveguide geometries.

## Introduction

Zinc oxide (ZnO) is a well-known II–IV group wide-bandgap semiconductor (*E*_g_ = 3.37 eV), possessing a hexagonal wurtzite-type (sp. gr. *P*6_3_*mc*) structure with unit cell parameters *a* = 3.25 Å, *c* = 5.20 Å. In recent years, a lot of attention has been paid to the studies of ZnO nanostructures of various shapes, including oriented nanorods (NRs), nanowires, nanobelts, nanoparticles, etc. for versatile photonic applications [1–2]. ZnO NRs are especially attractive for short-wavelength nano-devices due to their high exciton binding energy (60 meV) [3], allowing for efficient excitonic photoluminescence at room temperature, and due to their good mechanical and thermal stability. Arrays of ZnO NRs can be grown on various substrates (e.g., Si, Al_2_O_3_, glass) either by gas phase processes (e.g., by vapor–liquid–solid epitaxy, metal-organic chemical vapor deposition, pulsed laser deposition), or by wet-chemical processes (e.g., the hydrothermal method, electrochemical deposition) [4]. The hydrothermal growth of ZnO NRs is a relatively simple, versatile and low temperature process [5]. ZnO NRs are used in gas sensors due to the high sensitivity of ZnO to chemical environments [6], for light-emitting diodes due to the compatibility with the GaN technology and random lasing [7] (in the blue, around 0.390 µm), and in dye-sensitized solar cells [8].

The absorption and emission properties of the ZnO nanomaterials are physically related to the defects in the ZnO structure [9]. This is due to the relatively open structure of ZnO where the Zn^2+^ ions occupy half of the tetrahedral (*T*_d_) sites and all the octahedral (*O*_h_) ones are empty. The structure of ZnO is determined by alternating planes of tetrahedrally coordinated Zn^2+^ and O^2−^ ions stacked along the [001]-axis. Thus, there are many options to accommodate intrinsic defects (e.g., interstitial Zn^2+^ ions, Zn_i_, or O^2−^ vacancies, V_O_, which are the most widespread ones), as well as external dopants (e.g., divalent transition-metal ions M^2+^ entering into the *T*_d_ sites, where M = Co, Mn, Ni or Cu). The energy levels of the intrinsic defects are located between the valence and conduction bands and have different ionization energies ranging from ≈0.05 to 2.8 eV [10]. Various emissions (fluorescence) in the visible (green, yellow and red) originating from the defects in ZnO have been demonstrated [11].

Such properties of ZnO NRs make them interesting for potential applications in lasers. Recently, a number of nanostructures exhibiting broadband linear absorption (from the visible to near-IR) and ultrafast and broadband saturable absorption have been proposed as “fast” saturable absorbers (SAs) for lasers operating in the passively Q-switched (PQS) and mode-locked regimes. These include carbon nanostructures (e.g., graphene, graphene oxide, graphite nanoparticles, single-walled carbon nanotubes (SWCNTs)) [12–15], few-layer transition metal dichalcogenides (TMDs, e.g., MoS_2_, WS_2_ [16–17], black phosphorus (BP) [18]), and topological insulators (TIs, e.g., Bi_2_Te_3_, Sb_2_Te_3_ [19–20], graphitic carbon nitride (g-C_3_N_4_) [21]). In the PQS regime, such structures enable the generation of nanosecond pulses at high repetition rates (up to MHz) and they are attractive for compact laser designs (e.g., microchip or waveguide lasers) [22].

ZnO NRs have not been explored as saturable absorbers, yet. Singh et al. studied Mn^2+^-doped ZnO NRs grown from an aqueous solution on indium tin oxide (ITO) substrates which exhibited saturable absorption (optical bleaching) at 0.532 µm under ns-laser excitation [23]. This effect was ascribed to the defects promoted by the Mn^2+^ doping. Some studies revealed reverse saturable absorption (optical limiting) in ZnO thin films and NRs [24]. Zhu et al. studied ultrafast saturable absorption of multiwalled carbon nanotubes (MWCNTs) on quartz substrates beaded with ZnO nanoparticles [25]. This effect was demonstrated at 0.780 µm with femtosecond pulses. Ahmad et al. employed ZnO nanoparticles in a polymer thin film as a SA for an Er fiber laser. The absorption saturation experiment performed at 1.560 µm revealed a saturation intensity as low as 16 kW/cm^2^ for this material [26]. Loiko et al. studied the saturable absorption of Co^2+^-doped ZnO nanocrystals in a glass matrix at 1.540 µm, employing them as a SA for a bulk Er laser [27], however, the saturable absorption was mostly due to the Co^2+^ ions in *T*_d_ sites.

In the present work, we demonstrate the suitability of hydrothermally grown, oriented ZnO NRs as SAs for lasers emitting at 1 and 2 µm, for the first time, to the best of our knowledge. The preliminary results of this work were presented in a conference paper [28].

## Results and Discussion

### Structural study

**Figure 1:**
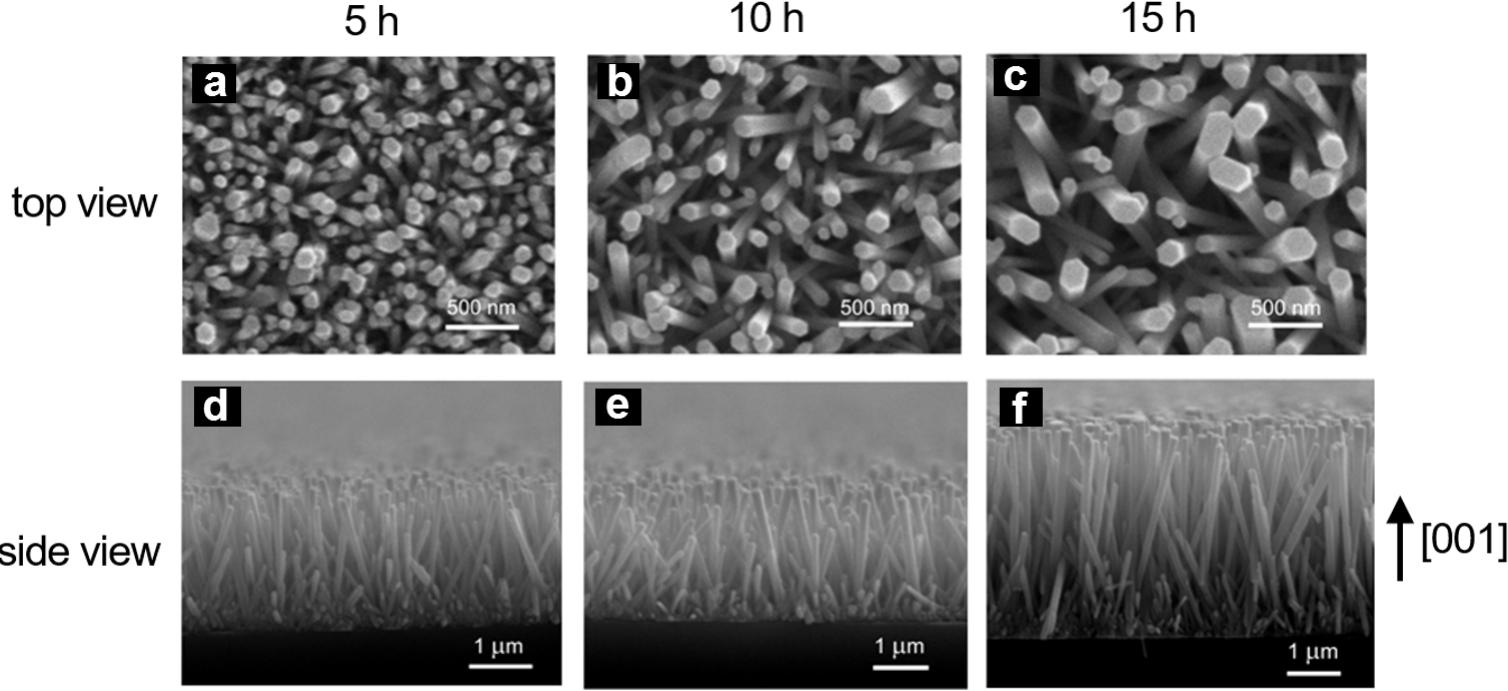
Field emission scanning electron microscope (FESEM) images of ZnO nanorods grown hydrothermally on glass substrates for (a,d) 5 h, (b,e) 10 h and (c,f) 15 h; (a,b,c) top- and (d,e,f) side-view. Images (b) and (e) are reproduced with permission from [28], copyright 2017, IEEE.

Figure 1 shows the field emission scanning electron microscope (FESEM) micrographs of the ZnO NRs grown hydrothermally for 5 h, 10 h and 15 h. The average length and diameter of the NRs were found to increase with the growth time, as summarized in Table 1. All ZnO NRs showed a characteristic hexagonal shape with orientation almost perpendicular to the glass substrate. In the initial growth periods, Ostwald ripening competes with the growth and results in merging of thinner NRs with thicker ones. With time, this process saturates and the growth continues. This is the reason why the growth of the NRs is not linear at the early stage of the process.

**Table 1:** Dimensions of the ZnO nanorods.

growth time (h)	avg. length (μm)	avg. diameter (nm)

5	2.1 ± 0.2	80 ± 20
10	2.4 ± 0.1	100 ± 25
15	3.5 ± 0.2	160 ± 25

**Figure 2:**
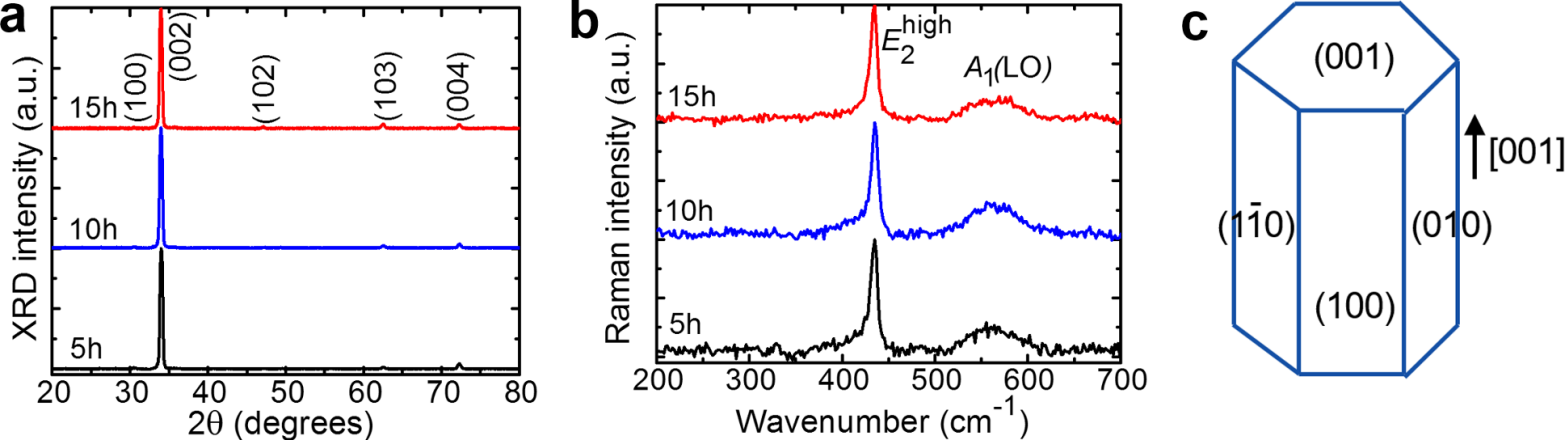
(a) X-ray diffraction (XRD) patterns and (b) Raman spectra measured with an excitation wavelength of 0.532 µm of ZnO NRs grown hydrothermally on glass substrates for 5–15 h; (c) schematic of the NR shape. The data for 10 h growth duration are adapted from [28].

Figure 2a shows typical XRD patterns of the ZnO NRs grown for 5–15 h. All ZnO samples showed characteristic XRD peaks (verified from JCPDS card No. 01-070-8070) confirming the hexagonal wurtzite structure (sp. gr. *P*6_3_*mc*). The strongest XRD peak observed at 34.35° indicated the preferential orientation of the nanorods along the [001] crystallographic axis. No notable variation in the XRD patterns of the NRs was observed with respect to growth time.

The Raman spectra of the ZnO NRs are shown in Figure 2b, where a strong Raman scattering at 435 cm^−1^ representing the *E*_2_^high^ phonon mode of ZnO is seen along with a broad peak centered around 574 cm^−1^ representing the *A*_1_(LO) mode [29]. All samples showed almost identical Raman spectra irrespective of their growth times or sizes. A schematic of the shape of ZnO NRs is shown in Figure 2c where the growth direction is indicated.

### Linear optical spectroscopy

**Figure 3:**
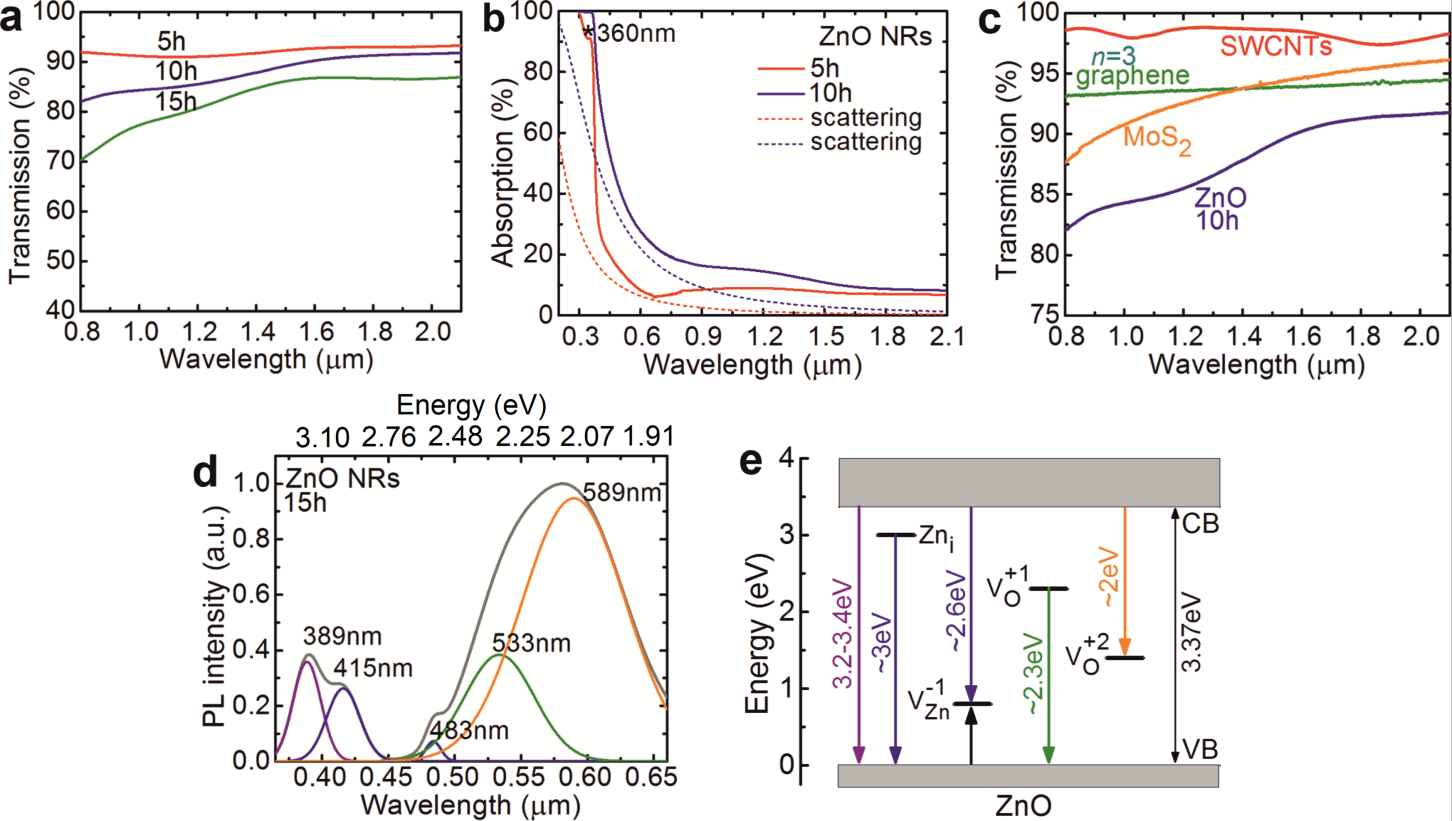
(a) Small-signal absorption spectra of the ZnO NRs grown hydrothermally on glass substrates for 5–15 h, *T*(ZnO NRs) = *T*(ZnO NRs + substrate)/*T*(substrate); (b) estimation of the scattering losses in ZnO NRs grown for 5 h and 10 h (dashed curves); (c) comparison of the small-signal absorption spectra of the ZnO NRs grown for 10 h, multilayer graphene with *n* = 3 carbon layers, multilayer MoS_2_ film [31] and SWCNT/PMMA film [32]; (d) room temperature photoluminescence (PL) spectrum of ZnO NRs grown for 15 h, the excitation wavelength is 0.350 µm: grey curve – the measured PL spectrum, color bands – decomposition of the PL spectrum into Gaussian components; (e) scheme of the defect states in the bandgap of ZnO (the color of arrows corresponds to the color of the emission bands in (d).

The results on the optical absorption and luminescence of the ZnO NRs are summarized in Figure 3. The small-signal (internal) absorption spectra of the ZnO NRs grown for 5–15 h are shown in Figure 3a. The spectra were corrected for the Fresnel losses arising from the substrate, *T*(ZnO NRs) = *T*(ZnO NRs + substrate)/*T*(substrate), where *T* is the measured transmission. With the increase of the growth duration, the transmission of the ZnO NRs decreases. Moreover, the scattering losses become more evident, as observed by the fast decrease of the transmission at wavelengths <1 µm, see also Figure 3b. Here, the scattering was modelled with an empirical formula (scattering loss ≈λ^−2.35^) [30]. The ZnO NRs are characterized by a broadband near-IR absorption featuring two broad bands centered at ≈1.2 and ≈2 µm, Figure 3a.

Let us discuss the nature of the broadband optical absorption of ZnO NRs. In Figure 3b for the NRs grown for 5 h, a characteristic absorption peak at ≈360 nm is observed (as indicated by an asterisk). This absorption peak is related to direct band-to-band transitions and its position is notably blue-shifted with respect to bulk ZnO due to the nanometer size effect. As the ZnO NRs were grown hydrothermally (in oxygen-rich conditions) one can expect the presence of zinc vacancies that have a low formation energy under such conditions. The singly charged zinc vacancy (V_Zn_^−1^) is typically located at 0.8–0.9 eV above the ZnO valence band (VB) [9,33–34]. This defect site can be a possible source for the near-IR absorption. For longer NRs, one can expect more V_Zn_^−1^ vacancies and stronger near-IR absorption, in agreement with Figure 3a.

In Figure 3c, we compare the small-signal absorption spectra of ZnO NRs grown for 10 h and several well-known nanostructured “fast” SAs, namely a commercial graphene-SA containing several (*n* = 3) carbon layers, a SA based on randomly oriented SWCNTs in a PMMA film [32], and a few-layer MoS_2_ SA [31]. One can observe a similar broadband absorption feature for the ZnO NRs as in these reference SAs.

The photoluminescence (PL) spectrum of ZnO NRs grown for 15 h is shown in Figure 3d. The excitation wavelength was 0.350 µm. The PL spectrum is composed of five major PL bands peaking at around 0.389, 0.415, 0.483, 0.533 and 0.589 µm.

The assignment of the PL bands of ZnO NRs is as follows. The band at 0.389 µm can be assigned to the direct recombination of electrons from the conduction band (CB) to the VB of ZnO (band-to-band transition). The violet emission near 0.415 µm can be assigned to the radiative recombination of electrons from a zinc interstitial (Zn_i_) defect level, typically located ≈0.22 eV below the CB, to the VB of ZnO [35]. The PL band at 0.483 µm is due to the radiative capture of an electron from the CB by a zinc vacancy state (V_Zn_^−1^) located around 0.8–0.9 eV above the VB of ZnO [9,33–34]. The broad green-yellow luminescence from the ZnO NRs is composed of two bands peaking at 0.533 and 0.589 µm. These bands are assigned to the singly (V_O_^+1^) and doubly charged (V_O_^+2^) oxygen vacancy states of ZnO, respectively [36–38]. Based on these assignments, a schematic diagram showing the position of the various defect states within the bandgap of ZnO and the corresponding PL lines is presented in Figure 3e.

### Absorption saturation

**Figure 4:**
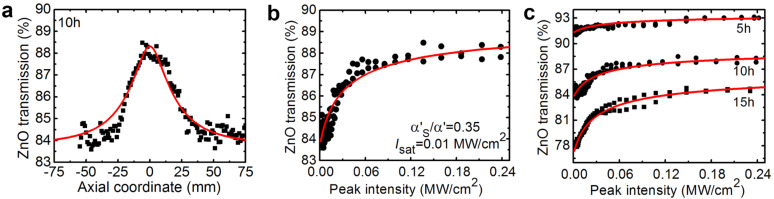
Absorption saturation of ZnO nanorods (NRs): (a,b) NRs grown for 10 h, (a) open-aperture Z-scan experiment and (b) the corresponding absorption saturation curve (measured at 1.06 μm under ps excitation), points – experimental data, red curve – fitting; (c) comparison of the absorption saturation curves for NRs grown for 5–15 h. The data plotted in (b) are adapted from [28].

The measured open-aperture Z-scan curve for the ZnO NRs grown for 10 h is shown in Figure 4a. It was fitted according to the formula α'(*I*) = α'_NS_ + α'_S_/(1 + *I*/*I*_sat_), where α' = 1 – *T* is the sample absorption (Fresnel losses were subtracted), *I* is the light intensity, α'_NS_ and α'_S_ is the non-saturable and saturable absorption, respectively, and *I*_sat_ is the saturation intensity. In the small-signal regime (*I* ≈ 0) the absorption is, α'_0_ = α'_NS_ + α'_S_ = 1 – *T*_0_. There are thus two free parameters, α'_NS_ and *I*_sat_. The spatial and temporal distribution of the laser intensity on the sample were accounted for [31]. Figure 4b shows the absorption saturation curve for the ZnO NRs plotted vs the peak on-axis laser intensity, *I*_0_ = 2*E*/(π*w*_L_^2^Δτ*), where Δτ* ≈ 1.06Δτ.

The absorption saturation curves for the ZnO NRs grown for 5–15 h are compared in Figure 4c. The corresponding absorption saturation parameters are listed in Table 2.

**Table 2:** Absorption saturation properties of the ZnO nanorods.

*T* (h)^a^	*I*_sat_, kW/cm^2^	α'_0_	α'_S_	α'_S_/α'_0_

5	13	8.7%	2.2%	0.25
10	10	16.3%	5.7%	0.35
15	9	23.0%	9.7%	0.42

^a^Growth time.

Now let us discuss the absorption saturation of ZnO NRs. With the increase of the growth duration from 5 to 15 h, *I*_sat_ decreases from 13 to 9 kW/cm^2^; the saturable absorption α'_S_ also increases from 2.2% to 9.7%. Moreover, the fraction of the saturable losses, α'_S_/α'_0_, increases from 0.25 to 0.42. This effect is assigned to the increased diameter of the NRs and their crystallinity and weaker boundary effects. The non-saturable losses of the ZnO NRs can be partially attributed to the scattering losses. It should be noted that the observed values of *I*_sat_ for the ZnO NRs are much lower compared, e.g., with the few-layer MoS_2_ SA (0.5 MW/cm^2^) [31] which can be due to the at least partial coupling of light inside the NRs thus enhancing the light–matter interaction. Previously, the absorption saturation of ZnO nanocrystals in a polymer film was studied [26] at 1.560 µm also with picosecond pulses resulting in *I*_sat_ = 16 kW/cm^2^, which is close to our observations.

### Passive Q-switching by ZnO nanorods

The laser experiments were performed with the NRs grown for 5 h, because they exhibited minimum insertion loss. Stable Q-switching was also observed for the NRs grown for 10 h, though the pulse characteristics were inferior. The experiments were performed in a microchip-type laser cavity in order to shorten the laser pulses due to the reduced cavity roundtrip time.

**Figure 5:**
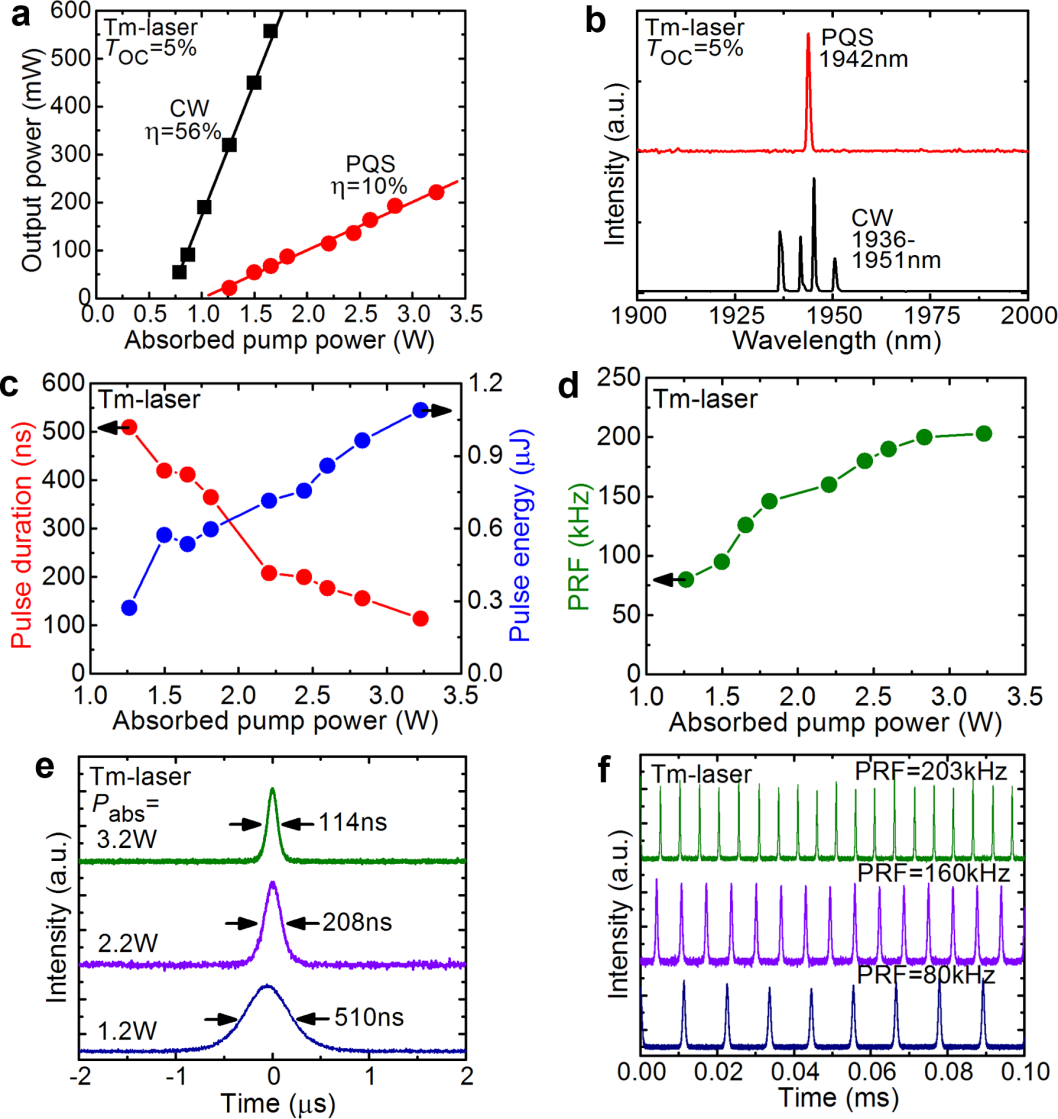
Tm:KLuW laser: CW operation and passive Q-switching by ZnO NRs grown for 5 h: (a) input–output dependences, η – slope efficiency (the data for PQS operation are adapted from [28]); (b) typical laser emission spectra measured at the maximum *P*_abs_; (c) pulse duration and pulse energy, adapted from [28]; (d) pulse repetition frequency (PRF); (e,f) typical oscilloscope traces of (e) single Q-switched pulses and (f) the corresponding pulse trains for different *P*_abs_ (the pulse train corresponding to a PRF of 203 kHz is adapted from [28]).

The performance of the Tm:KLuW laser PQS by the ZnO NRs was studied first. This laser generated a maximum average output power of 221 mW at 1.942 µm with a slope efficiency η of 10% (with respect to the absorbed pump power *P*_abs_), see Figure 5a,b. The laser threshold was at *P*_abs_ = 1.05 W. In the CW regime, the laser operated with higher efficiency, η of 56% and lower laser threshold of 0.70 W, so that the conversion efficiency with respect to the CW mode, η_conv_, was only 16% mostly due to the insertion losses of the SA. The emission spectrum of the CW laser consisted of several peaks in the spectral range 1.936–1.951 µm. These results correspond to the optimum transmission of the output coupler, *T*_OC_, of 5%. Power scaling of the laser in the PQS regime was limited by the Q-switching instabilities arising for *P*_abs_ > 3.2 W due to the heating of the SA by the residual (non-absorbed) pump [22].

The pulse duration Δτ, determined as full width at half maximum (FWHM) and the pulse repetition frequency (PRF) for the PQS laser were measured directly. The pulse energy *E*_out_ was then calculated as *P*_out_/PRF. These pulse characteristics for the Tm laser are plotted in Figure 5c,d. They show a notable dependence on the absorbed pump power, as expected for “fast” SAs. Indeed, Δτ decreases from 510 to 114 ns, while PRF increases from 80 to 203 kHz and *E*_out_ – from 0.3 to 1.1 µJ. Such a behavior is related to the increasing intracavity laser intensity on the SA and its effect on the SA dynamic bleaching [39]. The dependence of the pulse characteristics on *P*_abs_ is also illustrated in Figure 5e,f showing oscilloscope traces of single Q-switched pulses and the corresponding pulse trains. The single pulses have a nearly Gaussian temporal shape. The intensity instabilities in the pulse trains are <10%.

**Figure 6:**
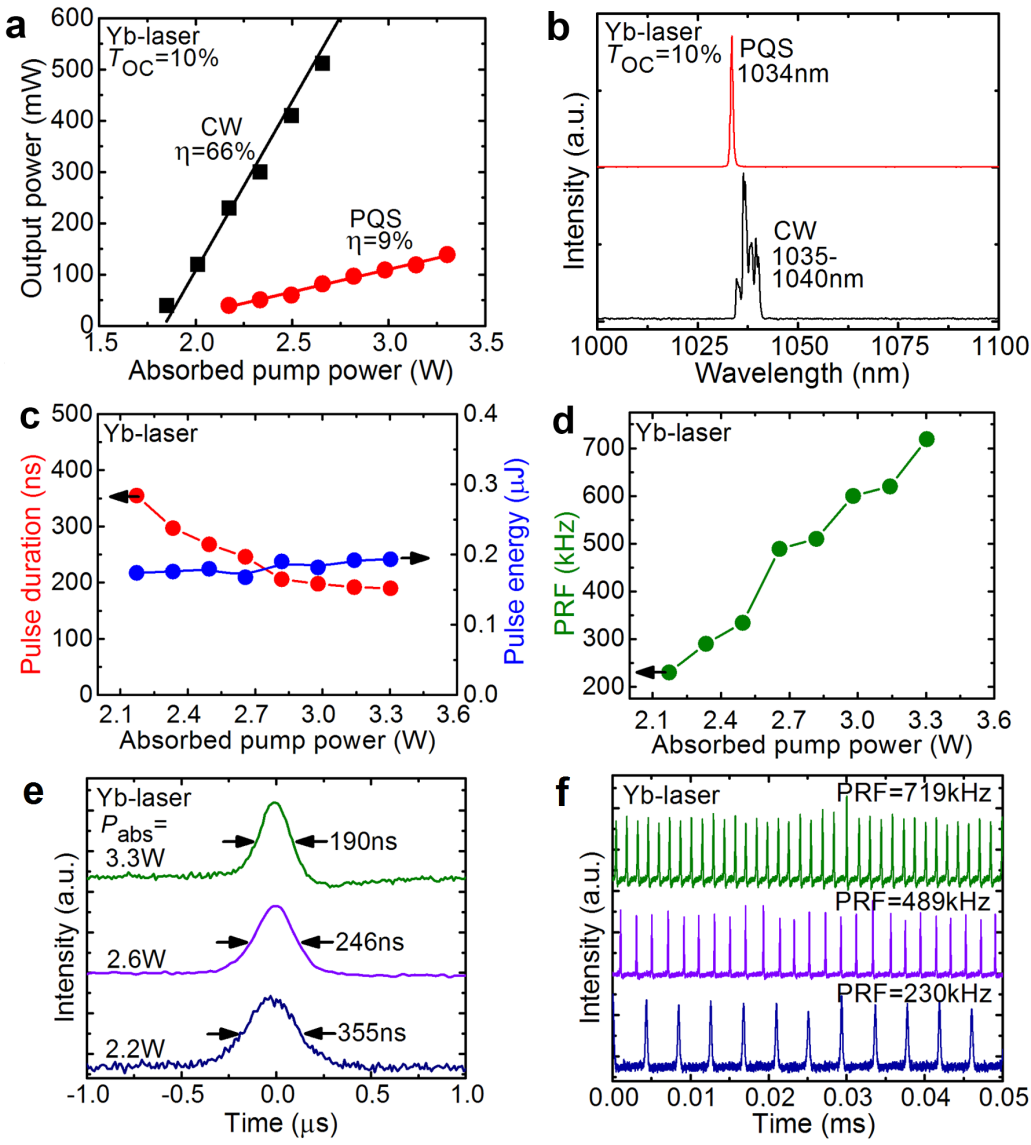
Yb:KLuW laser: CW operation and passive Q-switching by ZnO nanorods grown for 5 h: (a) input–output dependences, η – slope efficiency (the data for PQS operation are adapted from [28]); (b) typical laser emission spectra measured at the maximum *P*_abs_; (c) pulse duration and pulse energy; (d) pulse repetition frequency (PRF); (e,f) typical oscilloscope traces: (e) single Q-switched pulses and (f) the corresponding pulse trains for various *P*_abs_ (the pulse train corresponding to a PRF of 719 kHz is adapted from [28]).

A similar experiment was performed for the Yb:KLuW laser, Figure 6. In the PQS regime, this laser generated a maximum average output power of 139 mW at 1.034 µm with η = 9%, as shown in Figure 6a,b. The laser threshold was at *P*_abs_ = 1.90 W. In the CW regime, the laser operated with higher η of 66% and η_conv_ was 14%. In the CW regime, a multi-peak emission at 1.035–1.040 µm was observed. These results correspond to the optimum *T*_OC_ of 10%. The Q-switching instabilities were detected for *P*_abs_ > 3.3 W.

The pulse characteristics of the Yb:KLuW laser PQS by ZnO NRs (duration Δτ, PRF and energy *E*_out_), the oscilloscope traces of single Q-switched pulses and the corresponding pulse trains can be found in Figure 6c–f. With the increase of the absorbed pump power, the pulse duration decreases from 355 to 190 ns, the PRF increases from 230 to 719 kHz and the pulse energy was found to be weakly dependent on *P*_abs_ and amounted to ≈0.2 µJ. The pulse trains, Figure 6e,f, exhibit stronger intensity instabilities than those for the Tm laser.

### Comparison of ZnO NRs with nanostructured SAs

In Table 3, we compare the output characteristics of Yb:KLuW and Tm:KLuW lasers PQS by various “fast” SAs reported in the literature. These are graphene-SA based on single (*n* = 1) and several (*n* = 3) stacked carbon layers, few-layer MoS_2_ SA, a SA based on randomly oriented SWCNTs in a PMMA film (all of them deposited on a 1 mm-thick fused silica substrate), and a commercial transmission-type semiconductor saturable absorber (SESA, for Tm laser). For the Yb laser, the performance of all such nanostructured SAs is modest. In general, this is related to higher scattering losses in such SAs at ≈1 µm, as well as higher saturation intensities of such materials for higher photon energies. ZnO NRs are slightly better than such 2D materials as graphene-SA with a single carbon layer and few-layer MoS_2_. However, their performance is inferior as compared to the SWCNT-SAs, while the latter are less attractive since they are deposited in the form of a polymer thin film.

**Table 3:** Output characteristics of compact Yb:KLuW and Tm:KLuW lasers passively Q-switched by different “fast” saturable absorbers (SAs).

laser	SA	*P*_out_, mW	η, %	*E*_out_, µJ	Δτ, ns	PRF, kHz	ref.

Yb:KLuW	ZnO NRs	139	9	0.2	190	790	this work
	graphene (*n* = 1)	113	6	0.5	280	240	[40]
	graphene (*n* = 3)	315	12	1.0	140	320	[31]
	MoS_2_	147	7	0.5	220	300	[31]
	SWCNTs	633	17	1.1	80	602	this work
Tm:KLuW	ZnO NRs	221	10	1.1	114	203	this work
	graphene (*n* = 1)	310	13	1.6	285	190	[22]
	graphene (*n* = 3)	1030	39	4.0	190	260	[31]
	MoS_2_	1270	43	7.5	175	170	[31]
	SWCNTs	1360	41	5.5	48	250	[41]
	SESA	539	21	1.1	61	498	[41]

For the Tm laser, the best output characteristics are again achieved with the SWCNT-SA. The PQS performance of the ZnO NRs is better than other 2D materials (graphene-SA with a single carbon layer and few-layer MoS_2_) in terms of the pulse duration, similarly to the case of the Yb laser. This is attributed to the relatively large fraction of the saturable losses for the ZnO NRs, as summarized in Table 2.

ZnO nanocrystals (unoriented and with nearly-spherical shape) embedded in a polymer film have been previously applied as a SA only in Er and Yb fiber lasers. In the former case, the best pulse characteristics (duration/energy) were 3 µs/40 nJ corresponding to an output power of ≈3 mW at 1.561 µm [26]. The Yb fiber laser PQS by ZnO nanocrystals generated 1.6 µs/3 nJ pulses at 1.039 µm [42]. Thus, the present work, for the first time, demonstrates the ability of ZnO nanostructures to generate nanosecond pulses.

## Conclusion

Hydrothermally grown hexagonal-shaped [001]-oriented ZnO NRs are a promising material for broadband saturable absorbers for near-IR lasers (1–2 µm). In the present work, this has been verified by linear absorption measurements, the absorption saturation experiment at ≈1 µm and the application of the ZnO NRs in diode-pumped Yb- and Tm-doped lasers. The ZnO NRs exhibit low saturation intensity (about 10 kW/cm^2^ for ps pulses) which is two orders of magnitude lower than for 2D materials such as graphene and MoS_2_. The modulation depth of ZnO NRs can be easily adjusted by the growth duration.

The application of ZnO NRs as SAs in bulk Yb and Tm lasers indicated their capability to generate nanosecond pulses at high repetition rates up to the MHz-range with µJ-level pulse energy. The output characteristics of these lasers were limited by the scattering losses in ZnO NRs, which can be optimized by the synthesis method, for example, by additional annealing. Further improvement of the ZnO NR SA properties can follow two routes. First, the ZnO nanostructures can accommodate such transition-metal ions as Mn^2+^ or Co^2+^ in the *T*_d_ sites. This coordination is attractive for high ground-state transition cross-sections, so that the absorption saturation in the visible and near-IR can be observed. Moreover, the dopant ions can promote the absorption saturation due to the defects in the ZnO structure.

Second, the ZnO NRs can be hydrothermally grown directly on the crystal surface. This technology is especially attractive for the PQS waveguide lasers relying on the evanescent field interaction with the SA deposited on top of the active layer/channel. Taking into account the excellent sensing properties of the ZnO NRs, such composite structures can be of interest for the development of bio-molecule and gas sensors based on pulsed waveguide lasers.

## Experimental

### Growth of ZnO NRs

**Figure 7:**
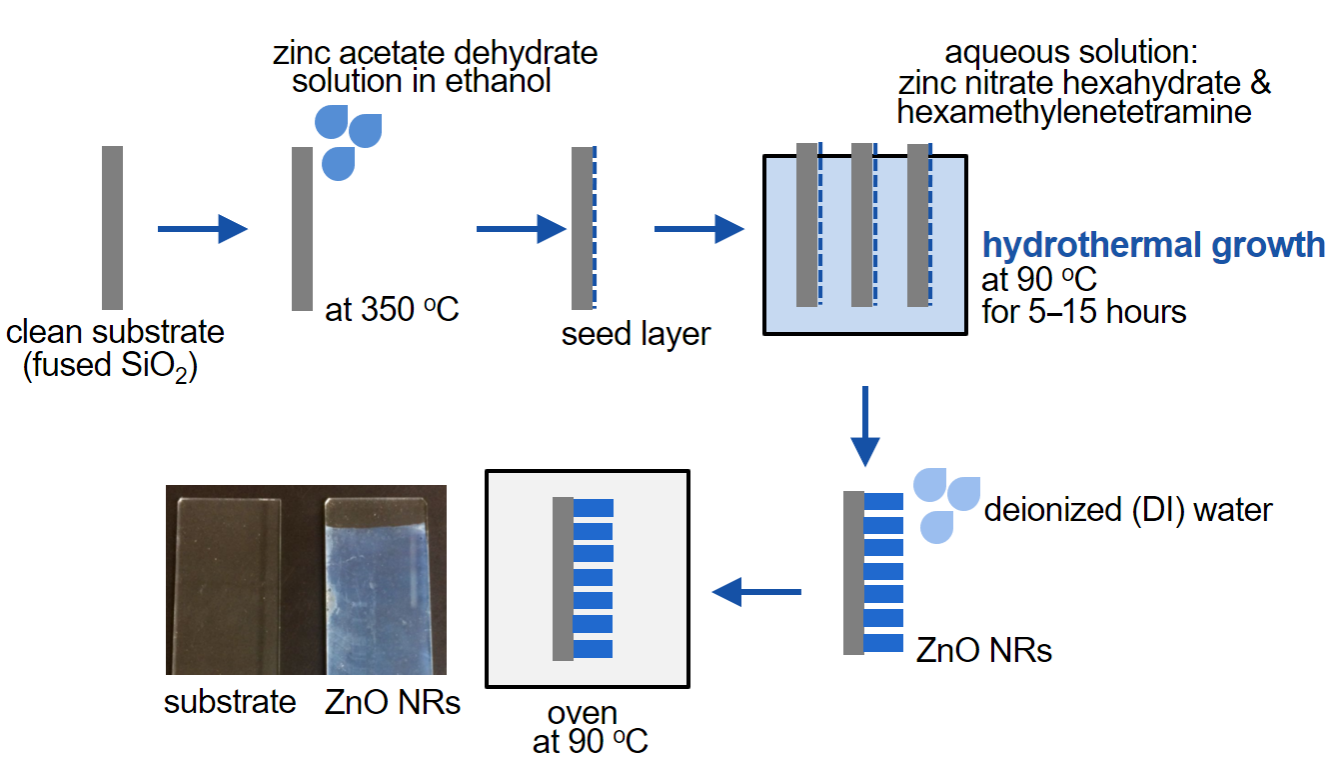
Schematic of the fabrication process of the ZnO NR saturable absorbers.

ZnO NRs were directly grown on microscope glass slides (thickness: 1 mm, double-side polished) using a hydrothermal process [43]. A ZnO seed layer was initially deposited on cleaned glass slides by spraying 5 mM zinc acetate dehydrate solution in ethanol at 350 °C. The role of this seed layer was to provide nucleation sites for the subsequent growth of ZnO NRs. The seeded substrates were then immerged in an aqueous solution of zinc nitrate hexahydrate (10 mM) and hexamethylenetetramine (10 mM) and the reaction bath was maintained at 90 °C for 5–15 h. During the hydrothermal growth, the reaction solution was replenished every 5 h in order to maintain a constant growth rate of the NRs. Finally, the ZnO-NR-coated glass slides were rinsed thoroughly with deionized (DI) water and dried at 90 °C in an oven. The fabrication process is schematically illustrated in Figure 7. The samples with ZnO NRs were white in color.

### Characterization of the nanorods

The morphology of the grown ZnO NRs was studied by field emission scanning electron microscopy (FESEM) using a JEOL JSM-7600F microscope. The structure of the grown ZnO NRs was studied by X-ray diffraction (XRD) using a Rigaku MiniFlex600 diffractometer. The XRD patterns of the samples were obtained using Cu Kα radiation (1.5406 Å) with diffraction angle from 20° to 80°.

The vibrational properties of the NRs were studied by Raman spectroscopy using a XploRA confocal Raman microscope from Horiba. The Raman spectra were recorded using a 0.532 µm CW laser excitation with a 10× objective and 1800 lines/mm grating (spectral resolution better than 2 cm^−1^). An integration time of 30 s was used.

The absorption spectra were measured using a CARY 5000 spectrophotometer (Varian).

The absorption saturation of the ZnO NRs was studied by the open-aperture Z-scan method. As an excitation source, a mode-locked 1.064 µm Nd laser was used with the following pulse characteristics: energy, *E* = 10 pJ (the laser beam was attenuated), duration, Δτ = 2 ps, and repetition frequency 58.15 MHz. The laser beam was focused to a spot size 2*w*_L_ of 92 μm with a Rayleigh length of 6.23 mm.

### Laser set-up

As a gain material, monoclinic double tungstate crystals, KLu(WO_4_)_2_, doped with 3 atom % Yb^3+^ (Yb:KLuW) or 3 atom % Tm^3+^ (Tm:KLuW) were used. The crystals were grown by the top-seeded solution growth (TSSG) slow-cooling method. The laser elements were cut along the *N*_g_ axis of the optical indicatrix and were 3 mm-thick and uncoated. These elements were pumped by a fiber-coupled laser diode (an InGaAs one, emitting at ≈0.978 µm, for the Yb laser, and an AlGaAs one, emitting at ≈0.802 µm, for the Tm laser). The polarization of the laser emission was linear, ***E*** || *N*_m_, naturally selected by the anisotropy of the gain. More details about the laser set-up can be found elsewhere [31].
